# Neurobiological stress responses predict aggression in boys with oppositional defiant disorder/conduct disorder: a 1-year follow-up intervention study

**DOI:** 10.1007/s00787-017-0950-x

**Published:** 2017-02-08

**Authors:** Jantiene Schoorl, Sophie van Rijn, Minet de Wied, Stephanie H. M. van Goozen, Hanna Swaab

**Affiliations:** 1 Department of Clinical Child and Adolescent Studies, Leiden University, Wassenaarseweg 52, PO Box 9555, 2300 RB Leiden, The Netherlands; 2 Leiden Institute for Brain and Cognition, Leiden University, Leiden, The Netherlands; 3 Department of Adolescent Development, Research Centre Adolescent Development, Utrecht University, Heidelberglaan 1, PO Box 80140, 3508 TC Utrecht, The Netherlands; 4 School of Psychology, Cardiff University, PO Box 901, Cardiff, CF103AT Wales, UK

**Keywords:** Cortisol, Heart rate, Parent training, PMTO, Parenting practices, Oppositional defiant, Disorder, Conduct disorder

## Abstract

To improve outcome for children with antisocial and aggressive behavior, it is important to know which individual characteristics contribute to reductions in problem behavior. The predictive value of a parent training (Parent Management Training Oregon; PMTO), parenting practices (monitoring, discipline, and punishment), and child neurobiological function (heart rate, cortisol) on the course of aggression was investigated. 64 boys with oppositional defiant disorder or conduct disorder (8–12 years) participated; parents of 22 boys took part in PMTO. All data were collected before the start of the PMTO, and aggression ratings were collected three times, before PMTO, and at 6 and 12 month follow-up. Parent training predicted a decline in aggression at 6 and 12 months. Child neurobiological variables, i.e., higher cortisol stress reactivity and better cortisol recovery, also predicted a decline in aggression at 6 and 12 months. Heart rate and parenting practices were not related to the course of aggression. These results indicate that child neurobiological factors can predict persistence or reduction of aggression in boys with ODD/CD, and have unique prognostic value on top of the parent training effects.

## Introduction

Antisocial and aggressive behaviors emerge in childhood and often extend into adolescence and adulthood, with a high risk of co-occurring negative outcomes, such as delinquency, unemployment, and psychiatric disorders [3]. The developmental course of aggression varies per individual. Identifying factors that may be associated with the developmental course of aggression would enhance our understanding of childhood aggression and may provide information relevant for interventions.

One of the factors identified that contribute to the course of aggression is negative parenting practices [26]. Children’s behavior is directly affected by parenting; poor parenting can reinforce disruptive behavior, for example, by giving into requests of the child to avoid tantrums. Poor parenting practices have been associated with higher levels of delinquency and aggression [14, 28], especially that monitoring and discipline are important for child outcomes [26]. Interventions targeting parenting practices are indeed found to be effective in reducing aggression in children [12, 13, 18, 21, 22, 24, 39]. Core to these parent training programs is the idea that changing the behavior of the child asks for the social environment to react differently to the child’s behavior. However, success rates show that not all children with antisocial and aggressive behavior respond positively to parent training programs and there is great variability in the amount of change achieved [24, 39]. Individual characteristics might explain why some children persist in their antisocial and aggressive behavior and others sensitively respond to parenting style [42].

Thus, besides parental factors, child characteristics should be taken into account when predicting future antisocial and aggressive behavior. Studies have found evidence of atypical neurobiological characteristics in children with aggression [43]. Individual differences in the neurobiological system of children might also be very important in relation to the effectiveness of interventions in reducing aggression [42]. Low resting heart rate (HR) is the best replicated biological correlate of antisocial and aggressive behavior [25], which was recently again confirmed in another meta-analysis [30]. Studies on the predictive value of resting HR and the course of aggression show conflicting results; some found that children with disruptive behavior disorders with low resting HR showed less reductions in oppositional defiant disorder/conduct disorder (ODD/CD) symptoms after intervention, thus profited less from treatment, than those with higher resting HR [38], whereas others did not find resting HR to be predictive of changes in externalizing problems in children with ODD/CD who received treatment [40]. In meta-analyses, low HR has been found to be predictive of future antisocial and aggressive behavior in community samples [25, 30].

Another important neurobiological correlate is cortisol, the end product of one of the main stress regulating mechanisms, and the hypothalamic–pituitary–adrenal (HPA) axis [43]. In general, studies have found lower levels of cortisol reactivity to stressors in children and adolescents with aggression problems [9, 10, 29, 37, 44, 45]. Of particular, interest is that low cortisol reactivity to stress was found to be predictive of higher levels of aggressive behavior in school-aged boys in treatment for ODD/CD, indicating that cortisol non-responders to stress are more persistent in aggressive behavior than cortisol stress responders [41]. In another study with an ODD/CD sample receiving treatment, cortisol reactivity was not predictive of more externalizing problems [40]. It is hypothesized that restoring the physiological stress response of a child with ODD/CD to a typical reactive state may lead to less aggression and more socially positive behaviors due to more adequate emotional and cognitive appraisals of socially stressful situations [43]. In three recent studies, it was indeed found that in preschool children at risk for developing antisocial behavior [4, 23] and in school-aged children with ODD/CD [8], cortisol response can be positively affected by treatment, which in turn mediated a greater decline in aggression [23]. Although the study of Van de Wiel et al. [41] did not examine cortisol change, this study does indicate that responders to stress showed less aggression than non-responders during follow-up. Therefore, even if changes in the HPA-axis occurred due to treatment, beforehand, it could already be predicted by the HPA responsivity who would show more reductions in aggressive behavior. Recently, individual differences were also found in cortisol recovery levels after a stressor in children with ODD/CD [33]. Failure to recover after a stressor may indicate limited coping behaviors and thus difficulties in adapting to environmental challenges [16]. To our knowledge, cortisol recovery has not been investigated in relation to the longitudinal course of aggression.

Thus, it is very important to not only focus on parental factors but also on child neurobiological factors, which might be differentially related to the course of aggression in individuals. By investigating neurobiological factors next to the parental factors, we might be able to predict the course of aggression even better. The aim of this study was, therefore, to examine the relative contribution of individual neurobiological factors, i.e., resting HR and cortisol reactivity and cortisol recovery, and parental factors, i.e., parent training and negative parenting practices, in predicting the course of aggressive behavior over 1 year in boys with ODD/CD. It was hypothesized that the parent training would be effective in reducing aggression in the clinical intervention group. We also hypothesized that negative parenting practices would be positively associated with aggression levels and that resting HR, cortisol reactivity, and impaired cortisol recovery would be negatively associated with aggression levels. Finally, it was hypothesized that all parent factors would predict the course of aggression and that adding neurobiological factors to the model would result in a better prediction of aggression over the course of 1 year.

## Method

The current study was approved by the Medical Ethical Committee of Leiden University Medical Centre (LUMC). Prior to participation parents and boys who were 12 years signed an informed consent according to the declaration of Helsinki.

### Participants

Inclusion criteria for all boys were an IQ > 70, age between 8 and 12 years, and a diagnosis of ODD or CD on the DISC-IV interview (Shaffer et al. 2000). All boys, irrespective of group membership, were recruited at clinical health centers (*n* = 22), special education schools (*n* = 31), or regular elementary schools (*n* = 12). After recruitment and parental consent, the number of participants in the study was 65. Specific parental consent was obtained for the clinical intervention condition (*n* = 22) and clinical control condition (*n* = 43). There was one drop-out in the clinical control condition, resulting in a final subgroup of *n* = 42.

All boys met the criteria for an ODD diagnosis (DISC-IV). Four boys in the clinical intervention group and 17 in clinical control group also met the criteria for a diagnosis of CD, and other comorbid diagnoses are shown in Table 1. The clinical intervention group (*M* = 89.5, SD = 12.61) had a significantly lower IQ score than the clinical control group (*M* = 99.1, SD = 14.06), *t* = −2.70, *p* = 0.009 (see Table 1 for more descriptive statistics).

**Table 1:** Descriptive statistics of the clinical intervention and clinical control group (mean ± SD)

		Clinical intervention (*n* = 22)	Clinical control (*n* = 42)	*t/χ* ^2^	*p*
Demographics	Age	10.4 ± 1.19	10.3 ± 1.35	0.16	0.872
	IQ	89.5 ± 12.61	99.1 ± 14.06	−2.70	0.009
	Caucasian	64%	61%	0.06	0.804
Comorbidity	CD	18%	41%	3.26	0.071
	ADHD	68%	71%	0.07	0.787
	Anxiety	55%	62%	0.32	0.569
	Depression	9%	17%	0.69	0.408
	Other	23%	31%	0.48	0.487
Medication	Psychostimulants	32%	41%	0.46	0.497
	Atypical antipsychotics	0%	10%	2.23	0.135

*CD* conduct disorder; *ADHD* attention deficit hyperactivity disorder; *other,* e.g., eating, tic disorder

### Parenting training

The parents of boys in the clinical intervention group received PMTO, an evidence-based, structured intervention, designed to enhance five parenting skills: limit setting and discipline, monitoring and supervision, problem solving, positive involvement, and skill encouragement, to reduce and prevent further escalation of child problem behavior (for details, see [24, 26]). These skills were practiced extensively in approximately 20 individual sessions once a week, through role play and problem-solving discussions with PMTO-certified therapists. Integrity of the intervention is monitored throughout via checks of video samples of the sessions.

### Measures

*IQ* was measured with Vocabulary and Block Design, two subtests of the Dutch version [19] of the Wechsler Intelligence Scale for Children (WISC-IV) [46]. These subtests have been found to provide a good estimation of full scale IQ scores [32].

*Frequency of aggression* was measured with the Parent Daily Report (PDR) [5], a reliable and valid index of observable aggressive child behaviors [27]. First, parents filled in if any of the 34 behaviors of the checklist described their child in the past half year (yes or no). Then, they were called three times a week and asked if the behaviors that best described their child’s aggression during the past half year (the questions they had previously responded to with ‘yes’) occurred during the previous 24 h (yes or no). Mean scores of these three 24-h checklists were calculated.

*Aggressive behavior* was measured with the Teacher Report Form (TRF/6-18) [1]. We used the subscale ‘aggressive behavior’ to reflect aggression reported by teachers.

*Negative parenting practices* were measured with the three negative parenting practices subscales of the Alabama Parenting Questionnaire (APQ) [36]: ‘supervision and monitoring’, ‘inconsistent discipline’, and ‘corporal punishment’. Internal consistency and validity have been reported to be moderate to adequate, and test–retest stability has been reported to be good [7].

*Neurobiology* was measured with resting HR, cortisol reactivity, and cortisol recovery.

*HR* was assessed by a 24 bipolar channel Porti-system from TMSi (Oldenzaal, Netherlands) at a sample frequency of 512 Hz and with a pre-high-pass filter of 0.5 Hz. The skin was first cleaned with alcohol, and then, pre-gelled disposable ECG electrodes were attached on the chest (sternum-V6 lead). HR was measured in beats per minute and calculated with Acqknowledge version 4.3.1. Resting HR was measured for 3 min, whilst boys were sitting in a comfortable chair and watching a relaxing video.

*Salivary cortisol* was collected using a tube (0.5 ml) in which boys could spit (passive drool). Samples were collected in the afternoon during (reactivity) and after (recovery) an established and ecologically valid psychosocial stressor. Boys were led to believe that they were competing against a videotaped opponent of similar age and sex for best performance and a highly favored award, whilst they were led to believe that they were losing out on winning the computer task competition (for details, see [9, 33, 34, 45]). Cortisol reactivity was calculated by the area under the curve with respect to increase (AUCi) [31]. Cortisol recovery was calculated by subtracting the first and last cortisol measure during the 1 h recovery phase [20].

**Fig. 1:**
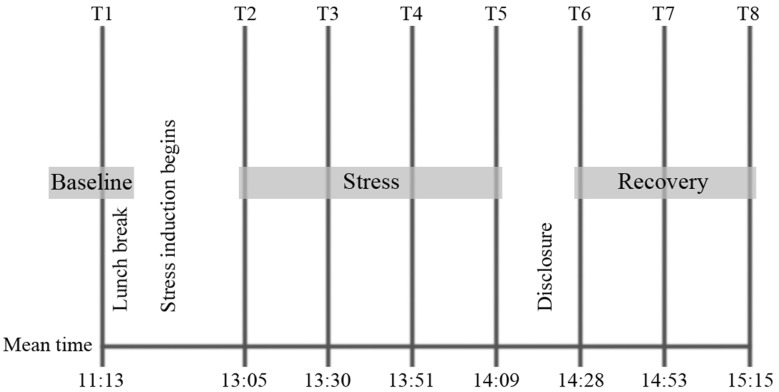
Schematic representation of the test procedure and mean sampling times. HR was only measured at T1. Cortisol was measured at T1–T8

Resting HR was measured before the psychosocial stressor began at time 1 (T1; see Fig. 1). Cortisol was measured eight times (T1–T8). The samples taken at T1–T5 were used to calculate cortisol reactivity (AUCi), and samples T6–T8 were used to calculate cortisol recovery.

### Design

The study consisted of three assessments over the course of a 12 month period: Time-1 was the pre-intervention measure when all variables were collected (cortisol, HR, APQ, PDR, and TRF). At Time-2, the post-intervention measure (approximately 6 months after Time-1), and Time-3, the 6 month follow-up (approximately 12 months after Time-1), parents and teachers reported again about the frequency of their child’s aggression (PDR) and aggressive behavior of the child at school (TRF).

### Statistical analysis

IQ was significantly higher in the clinical control group than the clinical intervention group (Table 1). A correlation analysis revealed that IQ was not related to aggression, and was, therefore, not controlled for in subsequent analysis.

First, we performed a repeated measures ANOVA (rANOVA) to compare the clinical intervention group and the clinical control group on their parent reported frequency of aggression and teacher reported aggressive behavior over time (Time-1, Time-2, and Time-3). A Greenhouse Geisser correction was applied if assumptions of sphericity were violated. If results were significant, we performed paired samples *t* test within each group to test if a significant reduction in aggression was present. Next, we performed a stepwise regression analysis to examine the relationships between parental factors, i.e., parent training and parenting practices, and neurobiological measures, i.e., HR and cortisol, as predictors and the course of aggression (frequency of aggression and aggressive behavior) as criterion. All regression analyses were performed within the larger group of boys with ODD/CD (*n* = 64). The course of aggression was calculated with delta scores (Δ) from Time-1 to Time-2, i.e., Δ short term, and from Time-1 to  Time-3, i.e., Δ long term. Effect sizes are reported as eta squared (*η* ^2^) with 0.01 being a small, 0.06 being a medium, and 0.14 being a large effect [6]. Cohen’s *d* effect sized was calculated for the paired samples *t* test with 0.2 being a small, 0.5 a medium, and 0.8 a large effect.

## Results

### Efficacy of the parent training

#### Frequency of child aggression as reported by parents (PDR)

**Fig. 2:**
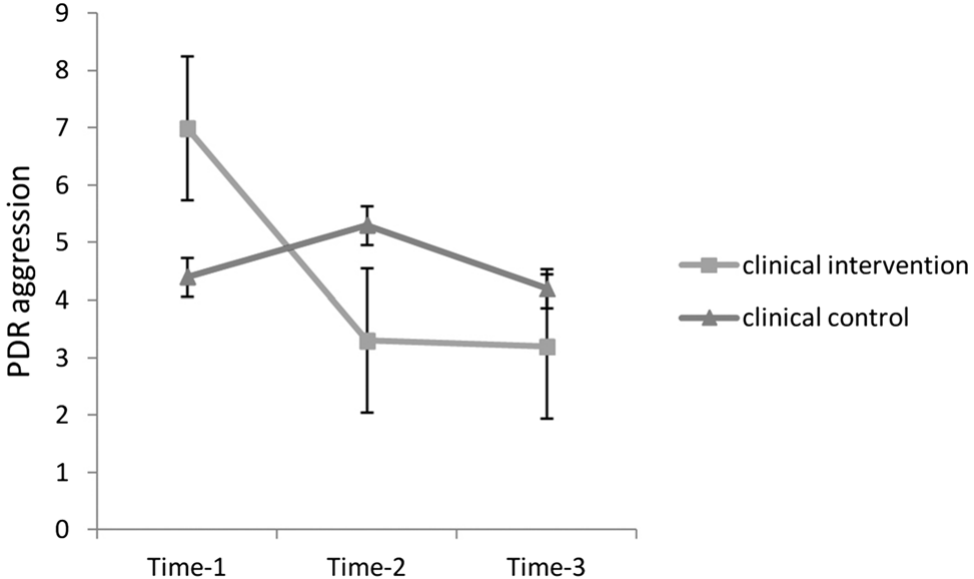
Mean and SE of parent rated frequency of aggression across 1 year in boys with ODD/CD

The rANOVA revealed that there was a significant main effect of time *F*(1.698, 81.482) = 8.16, *p* = 0.001, with a large effect *η* ^2^ = 0.15, and a time by group interaction, *F*(1.698, 81.482) = 10.49, *p* < 0.001, with a large effect *η* ^2^ = 0.18, but there was no main effect of group *F*(1,48) = 0.04, *p* = 0.845 (see Fig. 2). Post hoc paired samples *t* test revealed that the frequency of aggression was reduced in the clinical intervention group from Time-1 to Time-2, *t* = 4.15, *p* = 0.001, and *r* = 0.71, and from Time-1 to Time-3, *t* = 4.33, *p* = 0.001, and *r* = 0.73, whereas in the clinical control group, aggression rates did not change, *t* = −1.27, *p* = 0.211 and *t* = 0.53, *p* = 0.602, respectively.

Because of the higher frequency of aggression in the clinical intervention group at Time-1 compared to the clinical control group, *t* = 3.16, *p* = 0.002 (see Table 2), we performed another rANOVA with aggression frequency at Time-1 entered as a covariate. There was a significant main effect of group, *F*(1,47) = 6.94, *p* = 0.011, with a medium effect *η* ^2^ = 0.13, and there was a time by group interaction, *F*(2,94) = 5.65, *p* = 0.005, with a medium effect *η* ^2^ = 0.11, but there was no main effect of time, *F*(2,94) = 0.69, *p* = 0.504, indicating that frequency of aggression was significantly reduced over time in the clinical intervention group but not in the clinical control group.

**Table 2:** Mean and SD of aggression and parenting practices of boys with ODD/CD

		Pre-intervention (Time-1)	Post-intervention (Time-2)	Six month follow-up (Time-3)
PDR aggression (parent)	Clinical intervention	7.0 ± 2.78	3.3 ± 2.92	3.2 ± 3.34
	Clinical control	4.4 ± 3.12	5.3 ± 4.17	4.2 ± 4.08
TRF aggression (teacher)	Clinical intervention	18.1 ± 13.47	14.3 ± 10.99	11.5 ± 9.92
	Clinical control	14.7 ± 10.62	11.5 ± 8.21	12.1 ± 8.16
Parenting practices	Clinical intervention	8.2 ± 4.74	12.3 ± 2.64	0.9 ± 1.28
	Clinical control	7.07 ± 4.74	10.3 ± 2.80	1.0 ± 1.31

*PDR* parent daily report; *TRF* teacher report form; *missings Time-1* 5 parents, 6 teachers; *Time-2* 5 parents, 9 teachers; *Time-3* 6 parents, 10 teachers

#### Aggressive behavior rated by teachers (TRF)

The rANOVA revealed that there was a significant main effect of time *F*(2,84) = 4.46, *p* = 0.014, with a medium effect *η* ^2^ = 0.10, but not of group, *F*(1,48) = 0.75, *p* = 0.390, and there was no time by group interaction, *F*(2,84) = 0.71, *p* = 0.496. Although groups did not differ significantly from each other on aggressive behavior at Time-1 (see Table 2), we also performed a rANOVA with aggression frequency at Time-1 entered as a covariate for the aggressive behavior rated by teachers. The results remained the same, and there was an effect of time, *F*(2,82) = 4.76, *p* = 0.011, and *η* ^2^ = 0.10, but not of group, *F*(1,41) = 0.17, *p* = 0.686, or time by group interaction, *F*(2,84) = 0.94, *p* = 0.393.

### Predictive value of parental factors and neurobiological factors for the course of aggression

The correlation matrix shows that parent training was associated with greater decline in Δ shortterm and Δ long-term parent reported frequency of aggression (see Table 3). Inconsistent discipline correlated positively with greater decline in Δ short-term parent reported frequency of aggression. Cortisol recovery levels correlated positively with a greater decline in Δ short-term teacher reported aggressive behavior. No other correlations were found.

**Table 3:** Correlation matrix of parental and neurobiological predictors on aggression (*r*)

		PDR aggression		TRF aggression	
		Δ short-term	Δ long-term	Δ short-term	Δ long-term
Parent training		0.49***	0.38**	0.07	0.08
Parenting practices	Supervision/monitoring	0.12	−0.01	0.00	0.10
	Inconsistent discipline	0.27*	0.15	0.08	0.26
	Corporal punishment	−0.19	−0.16	−0.25	−0.19
Neurobiology	Resting HR	0.05	0.04	−0.23	0.06
	Cortisol/AUCi	0.04	−0.16	0.11	0.17
	Cortisol/recovery	0.12	−0.07	0.36*	0.16

*HR* heart rate, *AUCi* area under the curve with respect to increase, *Δ short-term* Time-1 − Time-2, *Δ long-term* Time-1 − Time-3

Missing cortisol: 11 boys were not able to produce saliva samples, missed one or more samples, or were inadequate for analyses, i.e., 3 SD above mean

* *p* < 0.05, ** *p* < 0.01, *** *p* < 0.001 (two-tailed)

We performed a stepwise regression analysis to predict the course of aggression. In step 1, we entered parental predictors, i.e., parenting training and parenting practices, i.e., monitoring, discipline, and punishment. In step 2, we added the neurobiological predictors, i.e., resting HR, cortisol reactivity, and cortisol recovery, to find out if they could help explain variance in aggression on top of parental factors. The short-term course of parent reported frequency of aggression (Time-1 − Time-2) was best predicted by the model with only parent training, *F* = 13.70, *p* = 0.001, *R* = 0.49 (see Table 4). In this model, parent training was associated with more reductions in aggression and explained 24% of the variance in Δ short-term aggression.

**Table 4:** Regressions of predictors on Δ short-term aggression and Δ long-term aggression

	Step		*b*	SE *b*	*β*
Δ Short-term PDR aggression (parent)	1	(Constant)	−5.23	1.68	
		Parent training	4.37	1.18	0.49**
Δ Long-term PDR aggression (parent)	1	(Constant)	−7.49	2.06	
		Parent training	3.63	1.19	0.42*
	2	(Constant)	−9.08	2.06	
		Parent training	4.72	1.22	0.55**
		Cortisol/AUCi	0.35	0.15	0.34*
Δ Short-term TRF aggression (teacher)	1	(Constant)	3.89	1.63	
		Cortisol/recovery	3.49	1.44	0.36*

Short-term PDR *R* ^2^ for step 1 = 0.24; Long-term PDR *R* ^2^ for step 1 = 0.15; *R* ^2^ for step 2 = 0.26; Short-term TRF *R* ^2^ for step 1 = 0.13

*PDR* parent daily report, *TRF* teacher report form, *AUCi* area under the curve with respect to increase, *Short-term* Time-1 − Time-2, *Long-term* Time-1 − Time-3

* *p* < 0.05, ** *p* < 0.001

The long-term course of parent reported frequency of aggression (Time-1 − Time-3) was best predicted by the model with parent training and cortisol reactivity (AUCi), *F* = 8.04, *p* = 0.001, and *R* = 0.53 (see Table 4). Adding reactivity to the model resulted in a significant change in explained variance, Δ*R* ^2^ = 0.11, *p* = 0.016. Those receiving the parent training and those with high levels of cortisol reactivity showed more reductions in Δ long-term aggression.

The short-term course of teacher reported aggressive behavior was best predicted by a model that included only cortisol recovery levels, *F* = 5.86, *p* = 0.020, *R* = 0.36 (see Table 4). In this model, more reductions in cortisol recovery were associated with more reductions in aggression and explained 13% of the variance in Δ short-term aggression.

The long-term course of teacher reported aggressive behavior could not be predicted by the variables.

Parenting practices and resting HR were not related to the course of aggression.

Finally, we explored the possibility of an interaction between the neurobiological child factors and the parenting practices. We calculated the interactions between these variables and included them in the regression models in step 3. The interaction variables did not predict aggression, and all models remained the same.

## Discussion

The aim of this study was to predict the course of aggression from parental factors, i.e., parent training and parenting practices (monitoring, discipline, and punishment), and neurobiological factors, i.e., HR and cortisol, in boys with ODD/CD.

First, we verified that the parent training resulted in a significant decline in children’s aggression post-intervention and at 6 month follow-up. Parents who took part in the parent training (PMTO) reported a significant decline in frequency of aggression post-intervention and at 6 month follow-up; parents of the clinical control children, who did not take part, reported no significant change in aggression.

The teachers of both groups of children reported a similar decline in aggressive behavior at 6 month follow-up, irrespective of whether the child’s family had received an intervention or not. Therefore, although the parent training seemed to have been effective in reducing aggression at home or in the perception of the parents, surprisingly the teachers of these children noted a similar and significant improvement in behavior in both groups over time. It is well known that parents and teachers often report differences in child behavior and this is because parents and teachers have different perspectives on aggressive problem behavior. In this study, teachers were asked to globally evaluate the child’s aggressive behavior over the last 6 months. Parents, on the other hand, had to report the occurrence of specific aggressive behaviors of their child three times per week. These different measures, therefore, might provide an answer as to why the results do not point in the same direction. Another possibility is that across the three measurements in time, the statistical phenomenon of ‘regression towards the mean’ may have occurred with respect to the teacher reports, with these becoming less extreme over time (which indicates a reduction in aggression).

Second, we investigated whether adding neurobiological factors to the parental factors might better predict the course of aggression in boys with ODD/CD. In accordance with the decline in perceived aggression by parents in the clinical intervention group, receiving the parent training was indeed predictive of a reduction in aggression in boys with ODD/CD from pre-intervention to post-intervention and from pre-intervention to 6 month follow-up. However, parenting practices were not predictive. Interestingly, key to the aim of this study, neurobiological factors were also predictive of the course of aggression in boys with ODD/CD. Specifically, a more pronounced cortisol stress response and a better cortisol recovery were predictive of stronger decline in aggression over time. Thus, adding neurobiological information on top of the parent training resulted in a better prediction of the developmental course of aggression.

These results indicate that those with a lower cortisol reactivity, i.e., ‘non-responders to stress’, have a worse prognosis in terms development of aggression over time. This result is in line with the study of Van de Wiel et al. [41], who found that low cortisol reactivity predicted more aggressive behavior in school-aged boys with ODD/CD. Interestingly, a weaker cortisol recovery response was also predictive of more aggressive behavior in our study. Thus, neurobiological factors could help predict future aggression. Boys with ODD/CD who responded less to stress and boys with ODD/CD who recovered less well after stress showed less reductions in aggression over the course of 6 months and 1-year follow-up. This profile of ‘non responding’ and ‘non regulation’ seems to be predictive of a worse outcome in terms of aggression on short-term and long-term notices. This might be important information for determining what intervention fits the individual profile best. Children showing this biological risk profile might be better treated with psychopharmacological interventions to alter the biological stress system than psychotherapeutic interventions, such as parent training programs [42]. Although the parent training was effective in decreasing aggression levels in the clinical intervention group as a whole, the intervention may be even more effective if we could adjust the intervention based on their neurobiological profile. For example, those who find it difficult to regulate after a stressor might need extra help in learning self-regulation strategies, so that they become able to deal with stressors and will not react, for example, with (reactive) aggression.

Resting HR was not related to the course of aggression in this study. HR is known to be the best correlate of antisocial behavior and predictive of persistence of antisocial behavior [25, 30, 38]. Our null finding is not unique. Van Bokhoven et al. [40] also found that resting HR did not predict changes in externalizing problems in children with ODD/CD over a couple of years. Future studies should further investigate if resting HR is able to predict the course of aggression, especially since HR is much easier to measure than cortisol reactivity or cortisol recovery in clinical settings.

As expected, the parenting practices were related to aggression, specifically inconsistent discipline. According to Patterson [26], discipline and monitoring are important in predicting behavioral problems in children. The parent intervention (PMTO), which is designed to improve these parenting practices, was indeed predictive of the course of aggression in this study. In a 1-year follow-up study, PMTO predicted greater ‘effective discipline’ post-intervention which in turn predicted a decline in aggression at 1-year follow-up [15]. Unfortunately, we did not measure the parenting practices post-intervention or at 6 month follow-up, so we do know if the parent training influenced parenting practices. Nevertheless, studies examining the effectiveness of PMTO have generally found that parenting practices improve after PMTO [11, 15, 17, 24, 26].

Another limitation of this study is that we also measured cortisol and HR only pre-intervention. Therefore, we do not know if the parent training influenced biological responses in the clinical intervention group and might have influenced the decline in aggression. Previous studies have reported that cortisol response can be positively affected by treatment [8] and that this change mediates a stronger decrease in aggression [23]. It is thought that restoring the physiological stress response of a child with ODD/CD to a typical reactive state may lead to less aggression and more socially positive behaviors, because the emotional and cognitive appraisal of socially stressful situations will be more adequate [43]. Another limitation is that our sample size is relatively small, especially the clinical intervention group. Therefore, we were not able to predict the course of aggression specific for the clinical intervention group. It would have been interesting if we could have replicated the study of Van de Wiel et al. [41], though our results are in line with hers. A final remark is that we, like many other studies examining ODD/CD, included only boys. Problems with aggressive and antisocial behavior are not unique to boys, and they have been found in girls as well (e.g., [2]). To what extent, the results of our study can be generalized to girls needs to be investigated first.

In conclusion, the results of this study indicate that child factors, in this case neurobiological characteristics that are mechanisms underlying aggressive behavior, provide important information about the risks and changes of persistence or reduction of aggression in boys with ODD/CD. Individuals with a neurobiological risk profile, i.e., those who are less stress reactive and/or who recover less well from stress, are more persistent in aggressive behavior compared to those who show typical stress regulation. The neurobiology of the child might, therefore, be an important predictor of the developmental course of aggression, independent of the impact of intervention on aggression. These results need to be replicated in larger studies, so that we might be able to develop the most optimal intervention for an individual with additional information based on their neurobiological profile.
